# Coexistence of gastrointestinal stromal tumor and inflammatory myofibroblastic tumor of the stomach presenting as a collision tumor: first case report and literature review

**DOI:** 10.1186/s13000-015-0413-y

**Published:** 2015-10-06

**Authors:** Hyeong Chan Shin, Mi Jin Gu, Se Won Kim, Jae Woon Kim, Joon Hyuk Choi

**Affiliations:** Department of Pathology, Yeungnam University College of Medicine, 170 Hyeonchung-ro, Namgu Daegu City, 705-703 South Korea; Department of Sugery, Yeungnam University College of Medicine, 170 Hyeonchung-ro, Namgu Daegu City, 705-703 South Korea; Department of Radiology, Yeungnam University College of Medicine, 170 Hyeonchung-ro, Namgu Daegu City, 705-703 South Korea

**Keywords:** Gastrointestinal stromal tumor, Inflammatory myofibroblastic tumor, Collision tumor

## Abstract

Collision tumors of the stomach are rare. We report on a case of a collision tumor consisting of a gastrointestinal stromal tumor (GIST) and an inflammatory myofibroblastic tumor (IMT) of the stomach in a 16-year-old female. A polypoid mass located in the distal body of the stomach was observed on abdominal computed tomography. Laparoscopic wedge resection of the stomach and 4d lymph node biopsy was performed. On gross examination, a protruding submucosal mass, measuring 4 × 3.5 × 2.5 cm in size, was detected. Histological examination showed two distinct GIST and IMT component presenting a collision tumor. The small nodular area, composed of CD117-positive spindle cells, was typical of GIST, and the adjacent larger area, composed of myofibroblastic spindle cells with prominent chronic inflammatory cells infiltrate, mainly lymphocytes and plasma cells, had a characteristic appearance of IMT. The 4d lymph node showed metastatic inflammatory myofibroblastic tumor. To the best of our knowledge, this is the first case of a collision tumor consisting of a GIST and an IMT arising in the stomach.

## Background

Collision tumor is defined as co-existence of two adjacent but histologically distinct tumors with no histologic admixture at the interface. The synchronous occurrence of two different tumors in the stomach has been frequently reported [1–5]. However, collision tumors in the same gastric area are uncommon. Collision tumors consisting of adenocarcinomas coexisting with gastrointestinal stromal tumor (GIST) [6–9], neuroendocrine tumor [10, 11], leiomyoma [12], and schwannoma [13] have been reported. Diagnosis of collision tumors can be a challenge [14].

To the best of our knowledge, no case of a collision tumor consisting of a GIST and an inflammatory myofibroblastic tumor (IMT) has been previously reported in English literature. Herein we report the first case of a collision tumor consisting of a GIST and an IMT arising in the stomach of a 16 year-old female.

## Case presentation

**Fig. 1:**
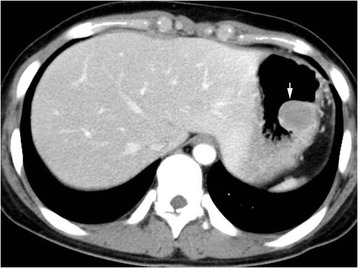
Abdominal CT scan. Abdominal CT scan shows a polypoid, submucosal mass (arrow) located in the distal body of the stomach

A 16-year-old female presented with abdominal pain for 2 weeks. Her medical and family history was nonspecific. Computed tomography of the abdomen showed a polypoid mass located in the distal body of the stomach (Fig. 1). Endoscopic ultrasound revealed a hypoechoic mass in the stomach. A polypoid mass located in the distal body with intact overlying mucosa was observed on esophagogastroduodenoscopy. The overlying mucosa was intact. The initial diagnosis was a gastric submucosal tumor. Laparoscopic wedge resection of the stomach and excisional biopsy of a 4d lymph node were performed.

**Fig. 2:**
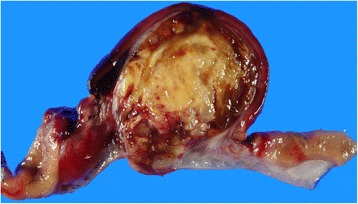
Gross finding. A well circumscribed, protruding mass is present in the distal body. The cut surface is yellow to red, and soft

**Fig. 3:**
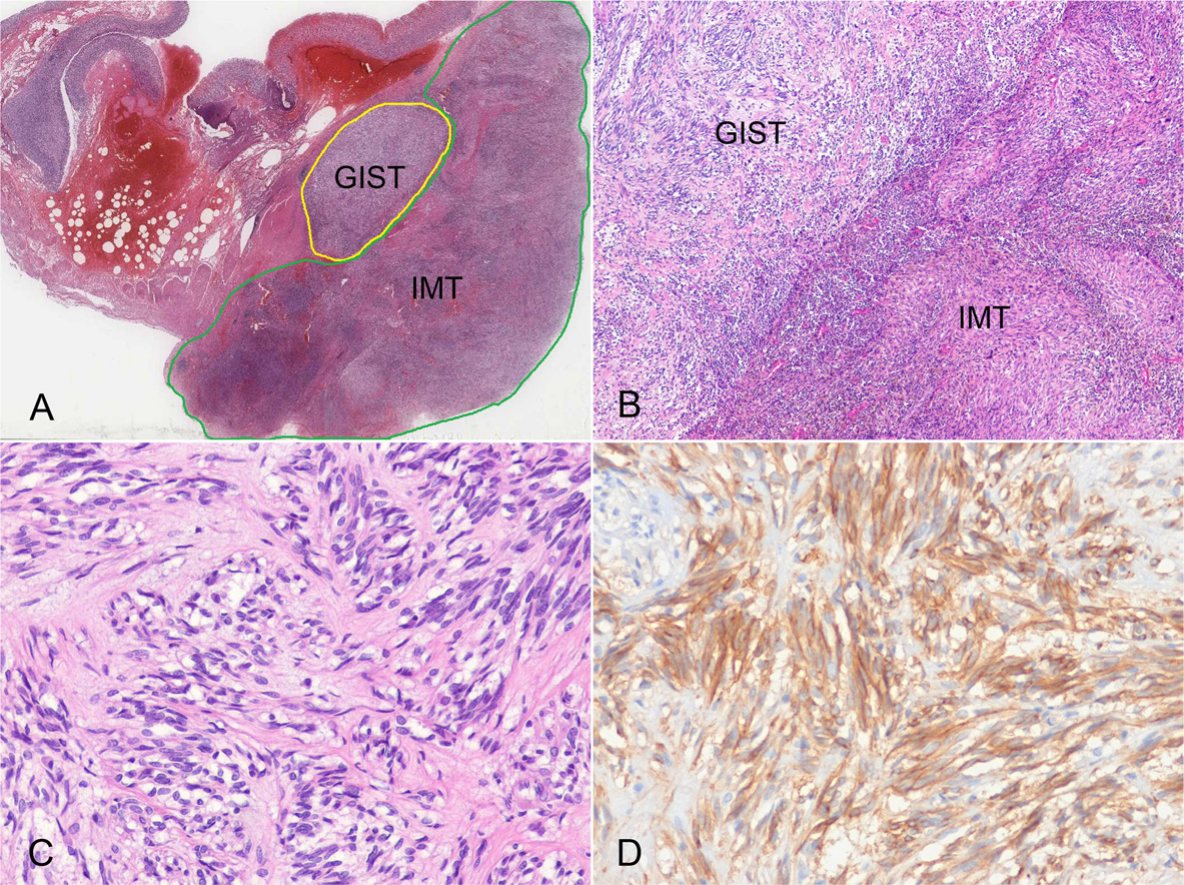
Histologic findings of gastrointestinal stromal tumor (GIST) area. **a** At low-magnification view, gastrointestinal stromal tumor (GIST) and inflammatory myofibroblastic tumor (IMT) area are mapped (H&E stain, x2). **b** GIST and IMT components are present in a collision tumor (H&E stain, x40). **c** Spindle-shaped cells with perinuclear vacuoles are present (H&E stain, x200). **d** The cells of the GIST area are positive for CD117 (Immunohistochemical stain, x200)

**Fig. 4:**
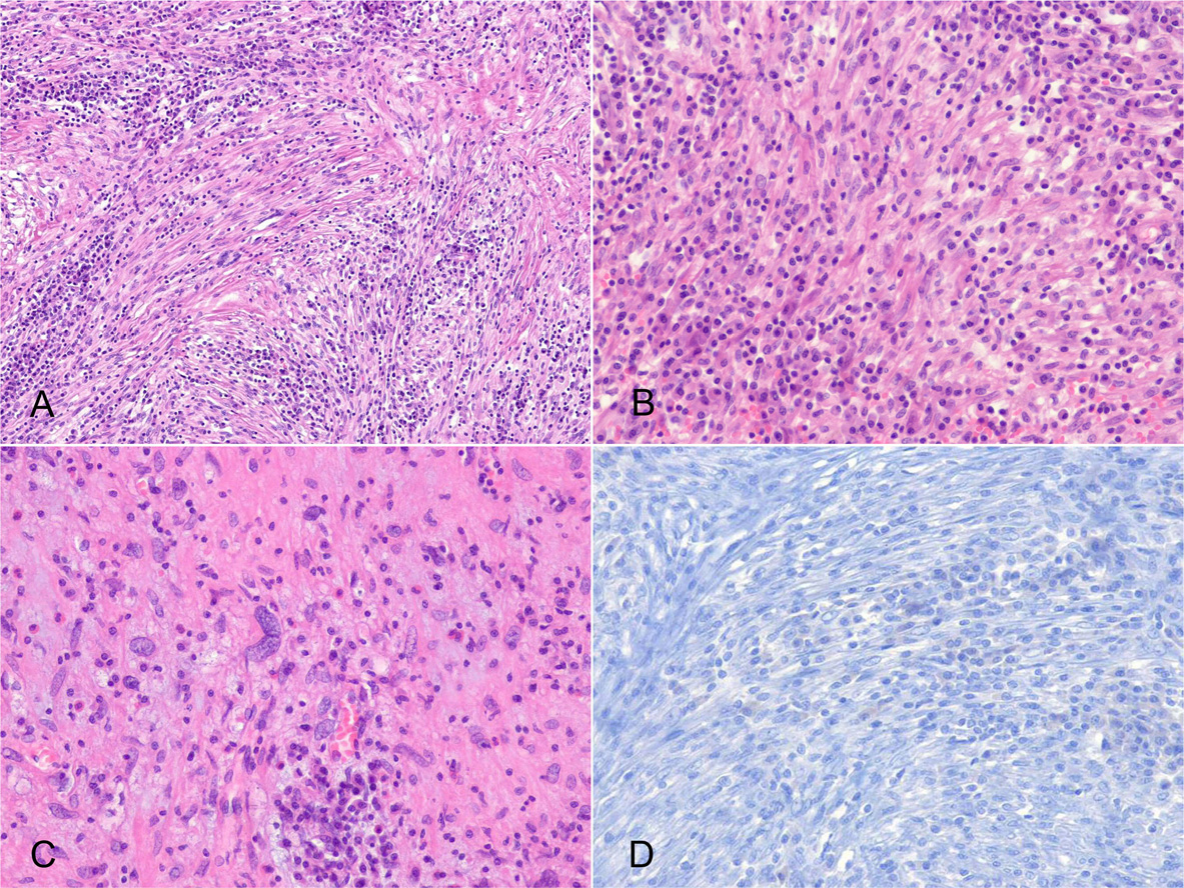
Histologic findings of inflammatory myofibroblastic tumor (IMT) area. **a** Myofibroblastic spindle cells are arranged in a fascicular pattern with prominent chronic inflammatory cells infiltrate (H&E stain, x40). **b** Spindle-shaped cells are admixed with lymphocytes and plasma cells infiltrate (H&E stain, x100). **c** The tumor cells have plump vesicular nuclei and eosinophilic cytoplasm. Collagenous stroma and scattered chronic inflammatory cells are present (H&E stain, x200). **d** The tumor cells are negative for ALK (Immunohistochemical stain, x200)

**Fig. 5:**
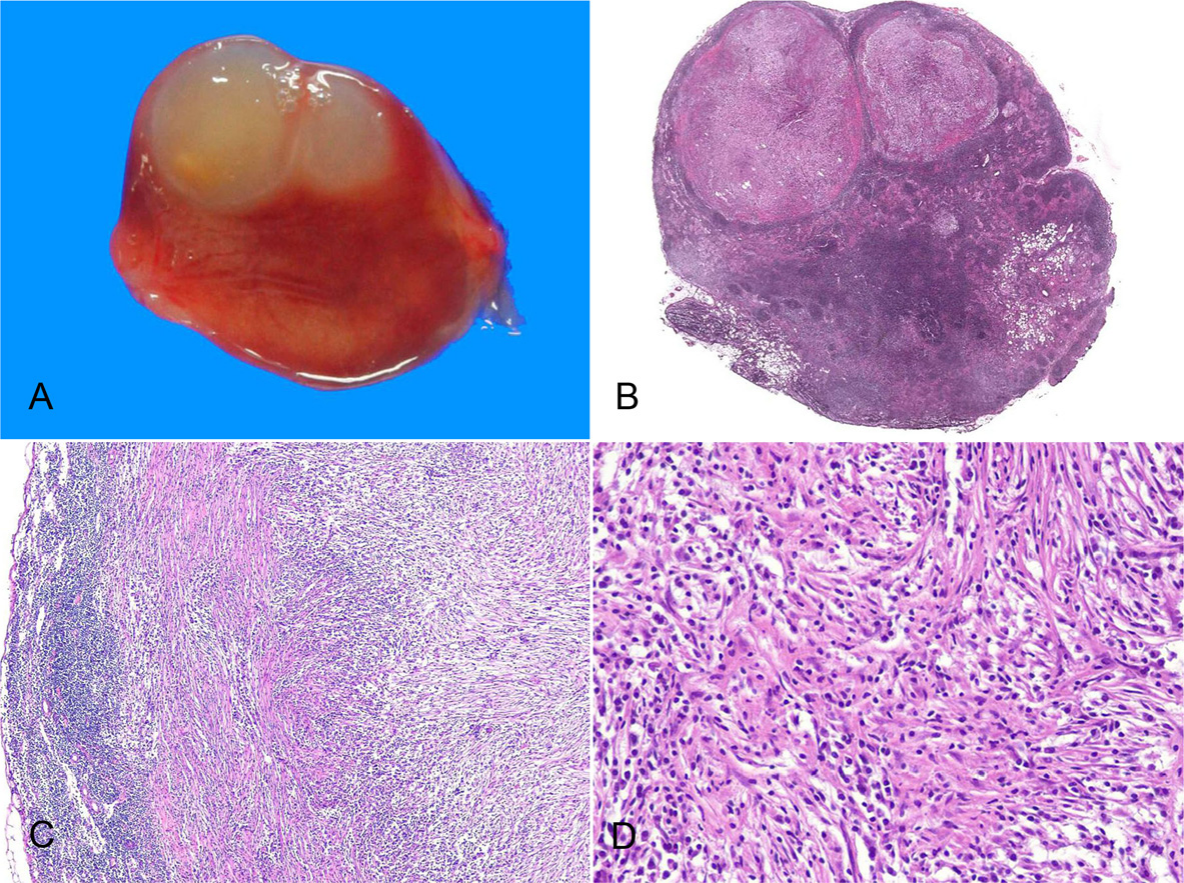
Gross and histologic findings of the 4d lymph node. **a** A gray-white, nodular lesion is located within the lymph node. **b** Whole mount section of the lymph node shows a nodular lesion (HE stain, x1). **c** Low magnification view shows replacement of the lymph node by tumor (HE stain, x40). **d** Spindle-shaped cells and lymphoplasmacytic infiltrate are present (HE stain, x100)

On gross examination, a well circumscribed, protruding submucosal mass was present, measuring 4 × 3.5 × 2.5 cm in size (Fig. 2). The cut surface was well circumscribed, yellow to red, and soft. Histologic examination of the mass showed the mass showed two distinct components in the muscularis propria (Fig. 3a, b). An area of palisaded spindle cells proliferation with uniform tapering nuclei and indistinct syncytial cytoplasm was observed. Perinuclear vacuoles were noted (Fig. 3c). Immunohistochemical staining showed that the cells of this component were positive for CD117 (Fig. 3d). The small area had characteristic appearance of GIST, spindle cell type, which measured 0.8 cm in size. The mitotic activity was 1 per 50 high-power fields (HPF). In mutational analysis for *KIT* performed from formalin-fixed and paraffin-embedded tissue, there were no *KIT* mutation in exon 9, 11, 13 and 17. The adjacent large area showed fascicular proliferation of myofibroblastic spindle-shaped tumor cells, admixed with prominent infiltrate of chronic inflammatory cells, mainly lymphocytes and plasma cells (Fig. 4a, b). The tumor cells had plump vesicular nuclei and eosinophilic cytoplasm (Fig. 4c). Some tumor cells had variably atypical nuclei. The immunohistochemical staining showed that the tumor cells of the large area were negative for CD117, DOG1, desmin, ALK, CD21, CD23, and S100 protein (Fig. 4d) and showed focal positivity for smooth muscle actin. In situ hybridization for Epstein-Barr virus (EBV)-encoded RNA did not show the presence of EBV. The majority of the infiltrating plasma cells were IgG-positive. The count of IgG4-positive plasma cells was less than 5 per high power field. Although the tumor cells were negative for ALK, the histologic appearance was characteristic of an inflammatory myofibroblastic tumor. A 4d lymph node showed a gray-white nodular lesion (Fig. 5a). Histologically, the lymph node was replaced by spindle cells proliferation and prominent lymphocytes and plasma cells (Fig. 5b, c, d). The spindle cells of the lymph nodes were negative for CD117 and ALK. The histologic findings of the lymph node were similar to those of the gastric IMT component. The lymph node was compatible with a metastatic inflammatory myofibroblastic tumor. The patient remains alive with no evidence of recurrence or metastasis 3 years after surgery.

## Discussion

Collision tumors of the stomach are rare. Gastric collision tumors composed of gastrointestinal stromal tumor (GIST) are shown in Table 1. Six cases of adenocarcinoma [6–9, 15], 1 case of signet ring cell carcinoma [16], and 1 case of angiosarcoma [17] showed collision with GIST. To the best of our knowledge, a collision tumor containing a GIST and an IMT has not been previously reported in English literature. Therefore, our case is the first case of a collision tumor containing a GIST and an IMT.

**Table 1:** Cases of gastric collision tumors composed of gastrointestinal stromal tumor in the literature

No.	Age/Sex	Collision tumors	Tumor location	Tumor size (cm)	References
1	70/M	Adenocarcinoma	Cardia and body	8 × 5 × 3	[6]
2	78/F	Adenocarcinoma,	Antrum	10 × 8	[7]
3	71/M	Adenocarcinoma	Esophagogastric junction	5 × 4 × 2.2	[15]
4	54/M	Signet ring cell carcinoma	Entire gastric wall	NA	[16]
5	73/F	Adenocarcinoma	Fundus and body	4 × 3	[8]
6	86/F	Adenocarcinoma	Minor curvature	6	[9]
7	78/M	Adenocarcinoma	Lessor curvature	6 × 5.5	[9]
8	81/M	Angiosarcoma	Anterior surface	9 × 5 × 4.5	[17]
9	16/F	IMT	Body	4 × 3.5 × 2.5	Present case

*IMT* inflammatory myofibroblastic tumor, *NA* not available

Gastrointestinal stromal tumor (GIST) is the most common primary mesenchymal tumor of gastrointestinal tract [18]. It is generally immunohistochemically positive for CD117 (KIT), phenotypically paralleling Cajal-cell differentiation, and in most cases contains *KIT-* or *PDGFRA-*activating mutations. Approximately 60 % of GISTs arise in the stomach. GISTs may coexist with different types of cancer, either synchronously or metachronously [14].

IMT is a distinctive lesion composed of myofibroblastic spindle cells accompanied by an inflammatory infiltrate of plasma cells, lymphocytes, and eosinophils [19]. It primarily affects children and young adults, although the age range extends throughout adulthood. It occurs throughout the body, most frequently in mesentery, omentum, retroperitoneum, pelvis, and abdominal soft tissue. Immunohistochemically, IMTs are generally actin positive and may also show staining for desmin and cytokeratin. Cytoplasmic reactivity for ALK protein is detected in 50–60 % of cases [19]. Therefore, ALK positivity is helpful in diagnosis of IMT, however, its absence does not exclude the diagnosis of IMT [20, 21]. Although our case was negative for ALK, the histological features were typical of IMT. Gastric IMT is rare and may be confused with other submucosal lesions [20]. IMT should be considered, particularly if the young patient has a gastric submucosal lesion showing spindle cells accompanied by numerous chronic inflammatory cells infiltrates, mainly plasma cells [22].

In the current case, presence of a GIST and an IMT in the same site resulted in formation of a collision tumor. No transition was observed between the different components of the tumors. We consider that the occurrence of two distinct tumors may be a simple incidental coexistence. Further investigation of the relationship between tumors of these types is needed.

The differential diagnosis of gastric IMT includes GIST, inflammatory fibroid polyp, smooth muscle tumors, schwannoma, IgG4-related sclerosing disease, and inflammatory pseudotumor-like follicular dendritic cell (FDC) sarcoma. GIST typically does not have the inflammatory background seen in IMT. The palely eosinophilic, syncytial cytoplasm and cytological uniformity of GISTs contrast with the plump myofibroblasts, scattered ganglion-like cells and collagenous background seen in IMT [21, 23]. Immunohistochemically, GIST is typically positive for CD117 but negative for ALK. IMTs are consistently negative for CD117. Inflammatory fibroid polyps (IFP) are usually present in the submucosa [24]. Histologically, there is proliferation of spindle and stellate stromal cells, which tend to condense around blood vessels to form whorled, perivascular cuffs, which are absent in IMTs. Both IFP and IMT have an inflammatory background, but that of the former is rich in eosinophils. The majority of IFPs are positive for CD34. Gastric smooth muscle tumors, which typically do not have an inflammatory background, have fascicles of spindle cells with cigar-shaped nuclei and brightly eosinophilic cytoplasm and are diffuse positive for smooth muscle actin, desmin and caldesmon. Gastric schwannoma shows peripheral cuff-like lymphoid aggregates and immunoreactivity for S-100 protein. IgG4-related sclerosing disease is a recently described multisystemic disorder with histological appearance similar to that of IMT [25, 26]. The prominent IgG4-positive plasma cells infiltrate is characteristic in IgG4-related sclerosing disease. In our case, the number of IgG4-positive plasma cells was low. The distinction of IMT from inflammatory pseudotumor-like FDC sarcoma may be difficult. The latter occurs almost exclusively in the liver and spleen [27, 28]. In the present case, no expression for FDC markers such as CD21 and CD23 can exclude inflammatory pseudotumor-like FDC sarcoma. Careful histologic evaluation, immunohistochemistry, and clinical correlation are helpful for a correct diagnosis of gastric IMT.

Due to their rarity, it is difficult to determine the biological behavior of gastric collision tumors. In our case, the GIST area belonged to the very low risk group. Risk of aggressive behavior and metastasis appears to be increased for ALK-negative IMT [21, 23]. The current case was negative for ALK and lymph node metastasis was present.

## Conclusions

We reported on a collision tumor consisting of a GIST and an IMT arising in the body of the stomach. This case is unique and the first report of a gastric collision tumor consisting of a GIST and an IMT. Awareness of this entity is important to distinguishing it from other submucosal lesions.

## Consent

Written informed consent was obtained from the patient for publication of this Case Report and any accompanying images. A copy of the written consent is available for review by the Editor-in-Chief of this journal.

## Abbreviations

GIST: Gastrointestinal stromal tumor
IMT: Inflammatory myofibroblastic tumor
IFP: Inflammatory fibroid polyp
